# Tendon regeneration and muscle hypotrophy after isolated Gracilis tendon harvesting - a pilot study

**DOI:** 10.1186/s40634-020-00236-8

**Published:** 2020-04-07

**Authors:** Anne Flies, Timm Denecke, Natascha Kraus, Philipp Kruppa, Matthew T. Provencher, Roland Becker, Sebastian Kopf

**Affiliations:** 1 Center for Musculoskeletal Surgery, Charité – University Medicine Berlin, Berlin, Germany; 2 Department of Radiology, Charité – University Medicine Berlin, Berlin, Germany; 3 Clinic and Outpatient Clinic for Orthopaedics and Orthopaedic Surgery, University Medicine, Greifswald, Germany; 4 Department of Plastic, Aesthetic and Reconstructive Microsurgery/Hand surgery, Hospital Ernst von Bergmann, Potsdam, Germany; 5 The Steadman Clinic, Vail, CO USA; 6 Center of Orthopaedics and Traumatology, Brandenburg Medical School Theodor Fontane, Hospital Brandenburg an der Havel, 14770 Brandenburg an der Havel, Germany

**Keywords:** Gracilis, Autograft, Tendon harvest, MRI, Radiologic outcome

## Abstract

**Purpose:**

The gracilis tendon (GT) is a commonly used autologous graft in Orthopaedic surgery. The majority of information on knee function and outcomes after hamstring harvest is related to both semitendinosus and GT harvest. Little is known regarding isolated harvest of a GT. It was hypothesized that isolated GT harvest would lead to altered gait patterns (e.g. augmented anterior-posterior translation or rotation in the tibiofemoral joint) and consequently a higher prevalence of cartilage lesions and meniscal tears in knees.

**Methods:**

GT harvesting was performed on patients with chronic acromioclavicular joint instability without previous knee injuries or surgeries. MRI of both knees and thighs were performed. Knee MRI were evaluated using the Whole-Organ Magnetic Resonance Imaging Score (WORMS). Inter- and intraobserver reliabilities, cross-sectional areas of different muscles, fatty infiltration of the gracilis muscle (GM) and GT regeneration were evaluated. The contralateral limb served as reference. The observers were blinded towards the identity of the patients and the operatively treated side.

**Results:**

After a mean time of 44 months after surgery testing was performed on 12 patients. No significant side-to-side differences were found using WORMS, although there was a trend towards increased cartilage lesions after GT harvest (median healthy knee 4.8 and GT harvested knee 7.8 *p* = 0.086**).** Inter- and intraobserver repeatability was high with 0.899 (95% confidence interval (CI) 0.708–0.960) and 0.988 (95% CI 0.973–0.995), respectively. A significant hypotrophy of the GM with a mean decrease of 25.3%, 18.4% and 16.9% occurred at 25% (*p* = 0.016), 50% (*p* = 0.007) and 75% (*p* = 0.002) of the length of the femur from distal. No compensatory hypertrophy of other thigh muscles or increased fatty infiltration of the GM was found. Tendon regeneration took place in eight out of 12 patients. In case of regeneration, the regenerated tendon inserted in a more proximal place.

**Conclusion:**

Isolated harvest of the GT for shoulder procedures did not affect knee MRI significantly indicating therefore in general suitable graft utilization for surgeries outside of the knee. GT regenerated in most patients with just a more proximal insertion and a hypotrophy of the muscle belly.

## Background

The gracilis tendon (GT) is a popular graft source for reconstructive procedures in orthopedic surgery [5, 17, 19, 29]. To our knowledge, the consequences of isolated GT harvesting in healthy knees remains unclear. Patients were only examined with torn ACL and reconstruction with isolated ST or combined ST and GT graft. Consequently, the outcome parameters were influenced by several factors: injury to the knee with damage of further structures (menisci, collateral ligaments), additional harvest of the ST tendon, surgery (ACL reconstruction), and rehabilitation of the knee. In studies on patients after rupture and reconstruction of the ACL with ST or ST and GT graft a substantially higher risk for developing osteoarthritis (OA) of the knee joint was shown [7, 18, 36]. Although it remains unclear whether development of OA was primarily because of the persisting rotational instability after ACL reconstruction [36] or also because of insufficient rotational control after harvest of hamstrings autograft [7]. The regenerative potential of ST and GT after harvesting for ACL reconstruction was for the first time reported by Cross et al. in 1992 [6]. Strength deficits were found after harvesting of the GT and ST tendon and as a consequence a compensatory hypertrophy of the hamstrings muscles [3, 10, 16]. But, it remains unclear whether deficits in thigh muscle strength were created by isolated harvesting of the GT. Only mild fatty infiltration was described after hamstrings tendon harvesting. It was hypothesized that further fatty infiltration did not take place because the functionality of the muscles was at least partially preserved [32, 37]. To sum up, the consequences of hamstrings tendon harvesting on the ipsilateral knee joint were analyzed by several studies. However, all of them presented a large number of factors influencing the outcome. Thus, the aim of the present study was to analyze the radiologic consequences of isolated GT harvest of otherwise healthy, uninjured knees.

As a primary outcome it was hypothesized that altered kinematics after GT harvesting would lead to a higher prevalence of cartilage lesions and meniscal tears in knees after GT harvest and therefore show a higher WORMS. As secondary outcomes it was hypothesized that after harvesting the GT, a tendon-like structure would regenerate, but lead to a hypotrophy and fatty infiltration of the gracilis muscle (GM). In addition, a compensatory hypertrophy of the other hamstrings muscles was hypothesized.

## Methods

In this retrospective cohort pilot study patients with chronic ACJ instability who underwent stabilization with autologous GT graft, in our clinic between 2007 and 2014, were considered for testing. The study was approved by the ethics committee of our institution (no. EA2/104/12). The study was registered at the German clinical trial register (no. DRKS00007100).

Written consent was given by all patients who met the inclusion criteria. Criteria for inclusion were: 1) stabilization of their ACJ with isolated GT at least 1 year before follow-up, 2) aged between 18 and 60 years, 3) no history of previous surgeries or injuries on both knees, and 4) ability to perform the MRI analysis (e.g. no claustrophobia).

Apart from the usual demographic data the Marx Activity Rating Scale was collected to describe our study population [20].

### Surgical technique

GT was harvested under general anesthesia and a single shot perioperative antibiotic. The tendon harvesting was performed by different well-experienced knee and shoulder surgeons. The patients were placed in the beach chair position because of the following intervention on the ACJ. After full relaxation a tourniquet was applied to the thigh. An approximately 2,5 cm longitudinal incision was made over the pes anserine. Then the sartorius aponeurosis was identified and an incision was made proximal to the GT over the length of approximately 4 cm. The GT was identified and released at its musculotendinous junction with an open tendon stripper. Finally the distal attachment was dissected and released from the bone [29].

### MRI examination

MRI of both knees and thighs were performed in dedicated multi-channel knee and surface coils using a 1.5 Tesla system (Avanto, Siemens, Erlangen, Germany*)*. Sequences allowing a good evaluation of the cartilage, menisci and ligaments were chosen for the knee and of the tendons and muscles for the thighs (Supplement 1). MR images were evaluated using the Osirix software (Pixmeo, Bernex, Switzerland).

**Fig. 1:**
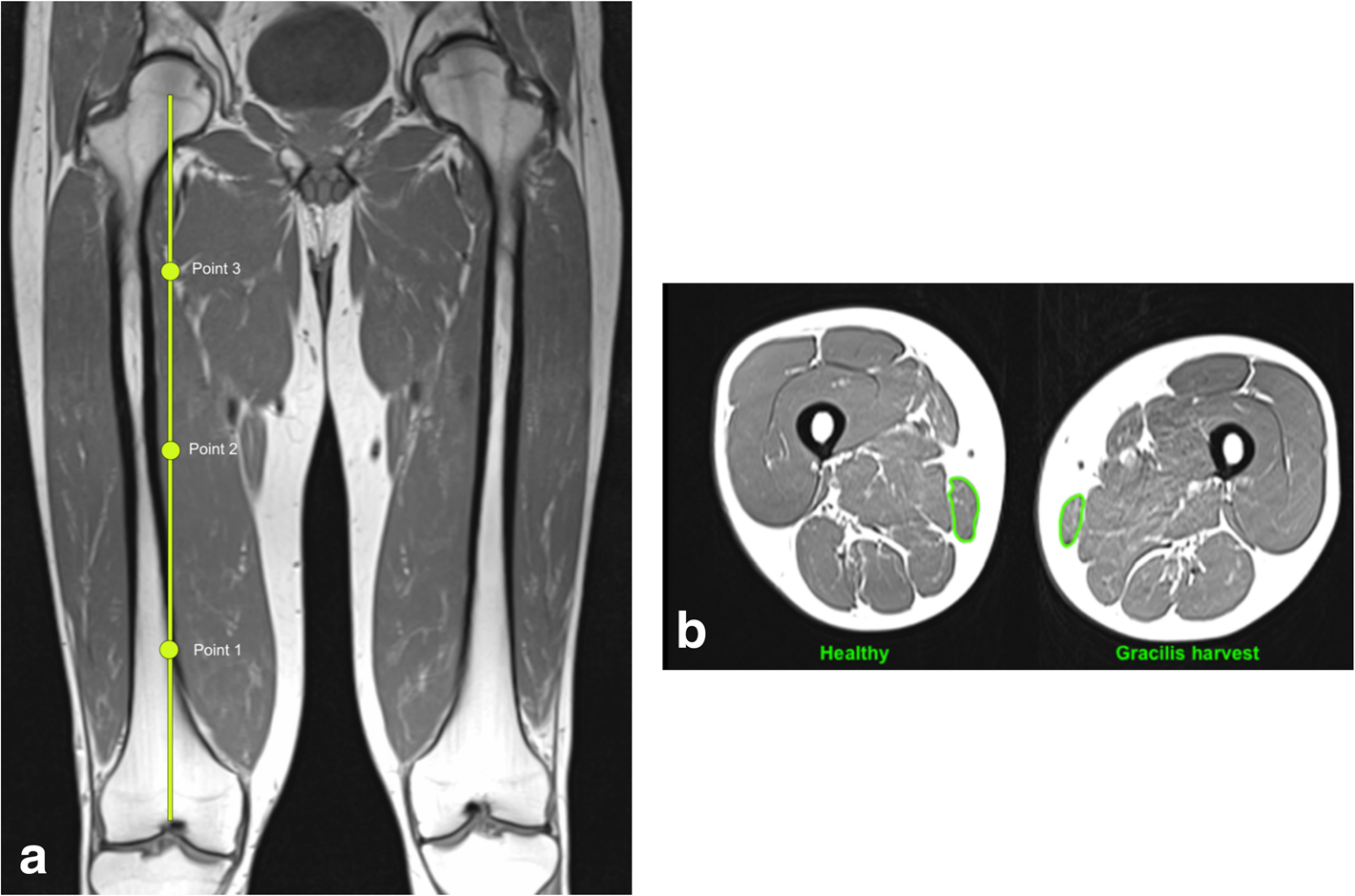
**a** and **b** Measurement of the CSA of the gracilis muscle. The bullets indicate 25%, 50% and 75% of the length of the femur from distal. Gracilis harvest = limb with previous harvest of the gracilis tendon, Healthy = contralateral limb

First, it was checked if the GT was harvested and not accidentally the ST. Knee MRIs were evaluated separately by three different researchers allowing the calculation of the interobserver repeatability. A second evaluation was performed 6 weeks later by the third researcher in order to calculate the intraobserver repeatability. The observers were blinded with regard to the identity of the patients and the operatively treated side. For the evaluation the Whole-Organ Magnetic Resonance Imaging Score (WORMS) was used [26]. Fourteen features of the knee joint were scored independently. Five of these features (cartilage, subarticular bone marrow abnormality, subarticular cysts, subarticular bone attrition, marginal osteophytes) were evaluated in 15 different regions (Table 1). Consequently, a total combined score as well as scores for the different features and regions were calculated. A healthy knee joint was scored 0. The higher the score the more abnormalities presented the knee joint. The worst possible score was 332. The contralateral healthy knee of each patient served as reference for the gracilis tendon harvested knee. Furthermore, a correlation analysis between the WORMS of the knee and time of follow-up was performed. In the T1-weighted axial images of the thigh, the cross-sectional area (CSA) of different muscles was measured at four different heights; the distal femoral growth plate, 25%, 50% and 75% of the length of the femur from distal. The CSA was measured using the closed polygon tool of OSIRIX (Fig. 1a and b, Table 2). In addition to CSA of the muscles, the fatty infiltration of the GM was evaluated as described by Engelken et al. [9]. These measurements were conducted using the ImageJ software [30]. Therefore, a slice at the transition from the middle to the distal third of the length of the femur in an axial t1-weighted sequence was chosen. First, reference measurements of pure fat and pure muscle tissue were performed in an oval region of interest (ROI) with approximately 200 counts. The intensity ranges in which all pixels represent pure fat or pure muscle were determined as the means ±2 standard deviations (SD). Next, the GM was defined as ROI with the freehand selection tool and intensity profiles were created for each pixel in this ROI. Then the pixels in the fat intensity and those in the muscle intensity range were added and a ratio (fat/muscle) was calculated. As reference, the same measurements were conducted at the contralateral thigh.

**Table 1:** WORMS features and regional subdivision of the knee joint. Features marked with * were evaluated in the different regions. Cartilage, bone attrition and osteophytes were not evaluated in the S-region

Features	Regional Subdivision	
articular cartilage integrity*	**Patellofemoral joint (PFJ)**	medial patella (MP)
subarticular bone marrow abnormality*		lateral patella (LP)
subarticular cysts*		anterior medial femoral condyle (MFa)
subarticular bone attrition*		anterior lateral femoral condyle (LFa)
marginal osteophytes*	**Medial tibiofemoral joint (MTFJ)**	posterior femoral condyle (MFp)
medial meniscal integrity		central medial femoral condyle (MFc)
lateral meniscal integrity		anterior medial tibial plateau (MTa
anterior cruciate ligament integrity		central medial tibial plateau (MTc)
posterior cruciate ligament integrity		posterior medial tibial plateau (MTp)
medial collateral ligament integrity	**Lateral tibiofemoral joint (LTFJ)**	central lateral femoral condyle (LFc)
lateral collateral ligament integrity		posterior lateral femoral condyle (LFp)
synovitis/effusion		anterior lateral tibial plateau (LTa)
intraarticular loose bodies		central lateral tibial plateau (LTc)
periarticular cysts/bursitis		posterior lateral tibial plateau (LTp)
	**Subspinous region**	portion of the tibial plateau beneath the tibial spines (S-region)

**Table 2:** Heights at which the cross-sectional areas of the muscles were measured from distal to proximal thigh. Empty fields indicate that the muscle was not identifiable

Height	Muscles						
	G	ST	SM	BF	Q	RF	VL/VM/VI
growth plate			x	x			
25%	x	x	x	x			
50%	x	x	x	x	x	x	x
75%	x	x			x	x	x

*G M. gracilis*, *ST* M. semitendinosus, *SM* M. semimembranosus, *BF* M. biceps femoris, *Q M. quadriceps*, *RF M. rectus* femoris, *VL/VM/VI* Sum of the Mm. vastus medialis, vastus lateralis and vastus intermedius

In addition, the observer checked whether the GT regenerated. It was distinguished between “no regeneration“, “tendon-like regeneration“, and “muscle-like regeneration “at the joint line, height of the distal femoral growth plate and at 25% of the length of the femur from distal.

### Statistical analyses

This was a pilot study, which is why an a priori power analysis was not performed but it was attempted to included all available patients operated at our institution. To test the Gaussian distribution of the population, the D’Agostino & Pearson omnibus normality test was used. To analyze for side-to-side differences the dependent t-test was used for parametric data and the Wilcoxon signed-rank test for non-parametric data. Parametric data are presented as mean and SD or 95% confidence interval (CI), whereas non-parametric data are presented as median and interquartile range (IQR). If applicable, the range was added e.g. for follow-up time and age. For correlation analyses the Pearson correlation coefficient was used for parametric data and otherwise the Spearman correlation coefficient. Predictive values were calculated as an approximation using r^2^.

The level of significance was 5% (*p* < 0.05) Intra- and interclass correlations were calculated using the Intraclass Correlation Coefficient (ICC) for absolute agreement [27]. All statistical analyses were performed using GraphPad Prism Version 6.01 (GraphPad Software, Inc., San Diego, CA, U.S.A.).

## Results

**Fig. 2:**
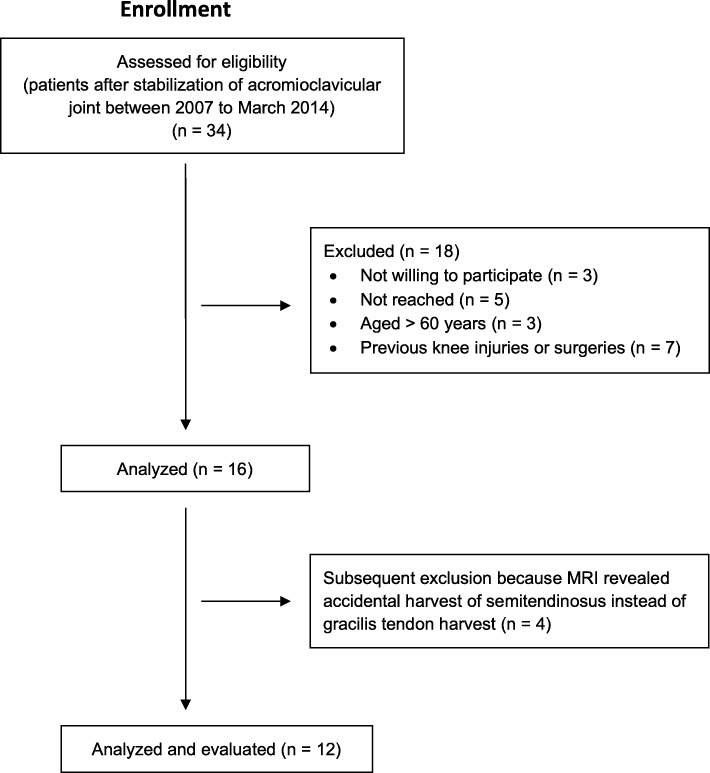
Flow chart illustrating patient enrolment

Sixteen patients were evaluated (14 male, 2 female). Retrospectively four patients had to be excluded because the MRIs showed accidental harvest of the ST tendon instead of the GT (Fig. 2). Demographic data is shown in Table 3. The gracilis tendon was harvested in equal parts from the right and left limb (six each).

**Table 3:** Demographic data

	Mean	Standard Deviation	Range
**Age** [years]	43	13	20–56
**Follow-up Time** [months]	44	25	20–93
**Body Mass Index** [kg/m^2^]	26.5	4.5	19.9–33.6
**Marx Activity Rating Scale** [points]	4	5	0–13

The total combined WORMS showed a median of 6.3 points (IQR 2.5 to 10) for the healthy limb and 10 points (IQR 5.4 to 15.5) for the limb with GT harvest (*p* = 0.095). The evaluation of the articular cartilage integrity of the knee joint showed a median of 4.8 (IQR 1.4 to 7.4) for healthy limbs and 7.8 (IQR 4.3 to 10.8) for limbs with GT harvest reaching almost statistical significance (*p* = 0.086). For all other features, statistical differences were higher (Table 4). Evaluation of the WORMS of the regions patellofemoral joint (PFJ), medial tibiofemoral joint (MTFJ) and lateral tibiofemoral joint (LTFJ) showed a median of 2.3 (IQR 0 to 3.8), 3.0 (IQR 0 to 3.9) and 0 (IQR 0) for the healthy limb and 3.3 (IQR 0.3 to 7.3), 3.5 (IQR 0.8 to 6.0) and 0 (IQR 0) for the GT harvested limb with a level of significance of *p* = 0.438, *p* = 0.234 and *p* >  0.05 respectively.

**Table 4:** Results of WORMS of the knee joint of healthy and gracilis harvested limbs

	Mean/Median Healthy	95%-CI/IQR Healthy	Mean/median Gracilis Harvest	95%-CI/IQR Gracilis Harvest	***p***-value
**Total combined score**	6.3	2.5–10	10	5.4–15.5	0.095
**Cartilage**	4.8	1.4–7.4	4.3–10.8	4.3–10.8	0.086
**Bone marrow abnormality**	0	0–0	0	0–0	0.375
**Subarticular cysts**	0	0–0	0	0–0	♦
**Subarticular bone attrition**	0	0–0	0	0–0	♦
**Osteophytes**	0	0–0	0	0–0	> 0.999
**Menisci**	0	0–0	0	0–2.5	0.125
**Ligaments (cruciates and collaterals)**	0	0–0	0	0–0	♦
**Synovitis/effusion**^a^	0.3	−0.1 - 0.5	0.3	−0.1 - 0.5	> 0.999
**Loose bodies**	0	0–0	0	0–0	♦
**Periarticular cysts / bursitis**	0.5	0.0–1.0	0.5	0.0–1.0	> 0.999

♦no difference between the two limbs, cannot calculate a paired t-test or Wilcoxon test. ^a^Gaussian distribution. *CI* confidence interval, *IQR* Interquartile range

Articular cartilage integrity in the PFJ, MTFJ and LTFJ showed a median of 1.5 (IQR 0 to 3.6), 3.0 (IQR 0 to 3.8) and 0 (IQR 0) for the healthy limb and 3.3 (IQR 0 to 4.8), 3.5 (IQR 0.8 to 6.0) and 0 (IQR 0) for the limb with GT harvest with a level of significance of *p* = 0.5, *p* = 0.141 and *p* >  0.05 respectively. For all other features and regions, no differences between the mean or median of the healthy and GT harvested limb were found.

Inter- and intraobserver repeatability was high with 0.899 (95% CI 0.708 to 0.96) and 0.988 (95% CI 0.973 to 0.995), respectively. A correlation between the total combined WORMS and time of follow-up could not be established (*p* >  0.05).

A tendon-like regeneration of the GT at the joint line was observed for five patients. At the height of the femoral growth plate, eight patients showed a tendon-like regeneration of the harvested GT. At 25% of the length of the femur from distal, a muscle-like regeneration was observed for four, a tendon-like regeneration for seven and no regeneration for one patient.

**Fig. 3:**
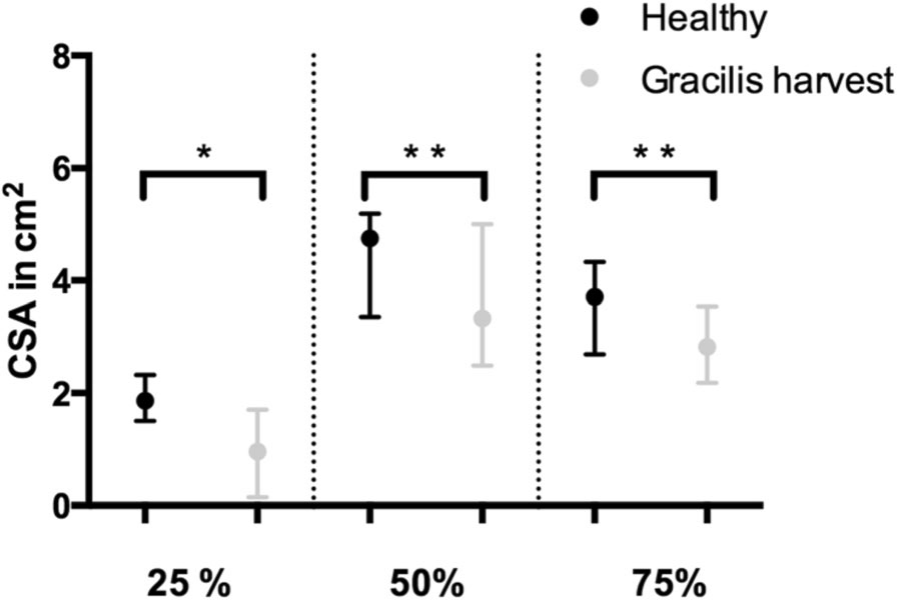
Comparison of the CSA of the gracilis muscle between limbs with gracilis tendon harvest (Gracilis harvest) and contralateral limbs (Healthy) at 25%, 50% and 75% of the length of the femur from distal. Results presented as median with IQR. * *p* < 0.05; * * *p* < 0.005

The CSA of the different muscles showed a statistically significant hypotrophy of the GM at all heights apart from the growth plate where the CSA on the operated side was not measurable because of the more proximal insertion of the tendon (Table 5, Fig. 3). The mean decrease of the CSA of the GM was 25.3%, 18.4% and 16.9% at 25%, 50% and 75% of the length of the femur from distal compared to the contralateral limb. A compensatory hypertrophy of the other thigh muscles was not observed, neither for all the patients nor in the subgroup where no regeneration of the tendon took place.

**Table 5:** Cross-sectional area of gracilis muscle

	Mean/Median Healthy (cm^**2**^)	95%-CI / IQR Healthy	Mean/Median Gracilis Harvest (cm^**2**^)	95%-CI/IQR Gracilis Harvest	***p***-value
**25%**	1.87	1.50–2.33	0.96	0.15–1.71	0.016*
**50%**^a^	4.54	3.76–5.31	3.70	2.68–3.76	0.007*
**75%**	3.71	2.69–4.33	2.82	2.18–3.54	0.002*

*reached statistical significance. ^a^Gaussian distribution. *CI* confidence interval, *IQR* Interquartile range

An increased fatty infiltration of the GM after harvesting of its tendon was not observed. The fatty infiltration of healthy GM was 0.006 (IQR 0.001 to 0.01), whereas it was 0.015 (IQR 0 to 0.028) (*p* >  0.05) for the ones with the harvested tendon.

## Discussion

The main finding of this study was that no statistical differences in the WORMS between the GT harvested thighs and the contralateral thighs were found. A trend towards increased cartilage lesions in the GT harvested thigh was shown in the WORMS. These lesions occurred especially in the PFJ and the MTFJ but without reaching statistical significance. The MTFJ and the lateral part of the PFJ represent also the regions in healthy knee joints with the biggest changes in cartilage thickness during loading and have therefore, irrespective of GT harvesting, a higher risk for developing OA [8]. Furthermore, changes in knee joint kinematics may be caused by harvesting of the GT and lead to early OA (e.g. smaller peak knee flexion angle, greater tibial rotation excursion, lower peak knee flexion moments) [7]. However, smaller knee flexion angles during stance phase of gait were also observed in uninjured patients with early patellofemoral OA because the surface through which patellofemoral loads are distributed is limited [7, 12]. Greater tibial rotation excursion after ACL reconstruction is potentially due to insufficient rotational control after hamstrings autograft and might be part of the mechanism after GT harvest too [7]. The small number of patients and the short time of follow-up might be an explanation for the missing statistical significance in the present study. However, a correlation between the total combined WORMS and time of follow-up was not found in the present study either. Thus, it remains unclear whether the tendency towards early cartilage lesions is a coincidence or due to tendon harvest.

High inter- and intraobserver repeatability in this study show good applicability of the WORMS in clinical use and the quality of the assessment.

Measurements of the cross-sectional area of the GM showed hypotrophy irrespective of regeneration of its tendon. However, no hypertrophy of the other thigh muscles, and especially the other hamstrings muscles, was seen. Hypotrophy of the GM in patients with GT and ST tendon harvest for ACL reconstruction was confirmed in the past [2, 15, 16, 28, 39]. On the other side, no decrease of the CSA of the G and ST muscle after harvesting for ACL reconstruction was found 10 cm above the joint, but this is distal to the main portion of the muscle bellies of most patients [31]. The findings of the present study contradict with prior published data showing a hypertrophy of the BF and SM muscles if no regeneration of the GT had occurred [3, 10, 14]. Hypertrophy of the SM muscle and long head of the BF muscle was even found irrespective of ST tendon and GT regeneration [16]. However, most of them harvested both, the ST and GT, tendons of muscles that have similar functions and therefore might lead to a higher deficit in muscle strength and a compensatory hypertrophy of the other hamstrings muscles. Findings of Eriksson et al. contradict this theory. After isolated harvesting of the ST patients without tendon regeneration showed hypertrophy of the SM muscle [10]. Factors that may implicate a compensatory hypertrophy of the SM and BF muscles are harvesting of multiple tendons (ST and GT) and if no regeneration of the harvested tendons takes place.

In the present study a fatty infiltration of the GM using MRI was not observed. Mild fatty infiltration (Goutallier Grade 1 or 2) of both ST and GM was shown in previous studies after harvesting both tendons in patients with ACL reconstruction [32, 37]. Histological analysis of the ST muscle of rabbits three, 6 and 12 months after harvesting their tendon ipsilateral showed no significant differences in fatty infiltration between both thighs (contralateral thigh was control). They hypothesized that low fatty infiltration was a sign that the muscle remained functional after tendon harvest [38].

Regeneration of the tendon of the GM took place in eight out of 12 patients. For these eight patients a tendon was detected at the distal femoral growth plate, but for only five of them a tendon was observed at the knee joint line, which lets us deduce that the regenerated tendons do not insert in their original localization, the *pes anserinus superficialis*, but in a more proximal position. This is in accordance with the findings of the majority of the previous studies after single ST tendon harvest or combined harvest of ST and GT. A more proximal insertion was shown in MRI [6, 28, 31, 34, 39], sonography [25] and surgical exploration [13]. Few authors, however, found in MRI or macroscopically during surgical exploration regenerated tendons at its normal insertion [1, 24]. Even a tibial insertion distal of the original point of insertion of the harvested tendon was detected, but with a more proximal musculotendinous junction [4]. Latter was confirmed by several authors [15, 21, 37]. It was hypothesized that the more proximal tibial insertion and musculotendinous junction had functional consequences and would lead to strength deficits especially for deep knee flexion angles (≥ 70°) [1, 4, 22, 23, 33, 35]. It was hypothesized that the more proximal tibial insertion and retraction of the muscle belly would lead to a shorter knee flexion moment arm and that therefore the concerned muscles are not able to produce the same amount of force [34, 35, 39]. At lower grades of knee flexion, the BF and SM muscles are the main producers of knee flexion strength and able to compensate here a loss of ST (and G) muscle strength [6, 39]. After ACL reconstruction with ST and GT autograft a correlation between the number of regenerated tendons and the strength deficit was shown [4]. Microscopically a fibrous structure appeared after 6 months, which evolved to a structure similar to the preharvest tendon after 2 years [11, 13], but some small areas with scar tissue persisted [13]. To summarize, scientific knowledge remains controversial if it comes to tendon regeneration and functionality of the regenerated tendons.

### Limitations

The present study has some limitations. First of all, more included patients would be favorable. Nevertheless, this was a pilot study looking for trends, where GT harvest might influence knee degeneration and muscle morphology of the thigh. All patients since starting with use of GT for chronic ACJ stabilization with a minimum follow-up of 1 year were tried to be included. Obviously**,** the risk of a type-II-statistical error was well-known. Unfortunately, the initial cohort was further decimated because of accidental harvest of the ST tendon.

A second limitation is the cross-sectional study design. Comparisons were only made between the surgical and contralateral limb. No presurgical comparisons existed and we had no data about the evolution of cartilage lesions, CSA of the thigh muscles and tendon regeneration over time. The present study cohort included only two female patients and therefore separate analyses on gender differences could not be performed. Some patients also presented short times of follow-up, which could make it difficult to detect degenerative changes or tendon regeneration. A correlation between the total combined WORMS and time of follow-up, however, could not be established. However, analysis of the radiologic consequences of isolated GT harvest in uninjured knee joints represent a novel and valuable addition to the literature.

## Conclusions

Isolated harvest of the GT for shoulder procedures did not affect knee MRI significantly indicating therefore in general suitable graft utilization for surgeries outside of the knee. GT regenerated in most patients with just a more proximal insertion and a hypotrophy of the muscle belly.

## Supplementary information

**Additional file 1: Supplement 1**. Sequences of the MRI of both knees and thighs

## Data Availability

The datasets used and analyzed during the current study are available from the corresponding author on reasonable request.

## Abbreviations

GT: Gracilis tendon
WORMS: Whole-Organ Magnetic Resonance Imaging Score
GM: Gracilis muscle
CI: Confidence interval
ACL: Anterior cruciate ligament
MPFL: Medial patellofemoral ligament
ACJ: Acromioclavicular joint
OA: Osteoarthritis
PFJ: Patellofemoral joint
MP: Medial patella
LP: Lateral patella
MTFJ: Medial tibiofemoral joint
MFa: Anterior medial femoral condyle
MFc: Central medial femoral condyle
MFp: Posterior medial femoral condyle
MTa: Anterior medial tibia plateau
MTc: Central medial tibia plateau
MTp: Posterior medial tibia plateau
LTFJ: Lateral tibiofemoral joint
LFa: Anterior lateral femoral condyle
LFc: Central lateral femoral condyle
LFp: Posterior lateral femoral condyle
LTa: Anterior lateral tibia plateau
LTc: Central lateral tibia plateau
LTp: Posterior lateral tibia plateau
CSA: Cross-sectional area
G: Gracilis
ST: Semitendinosus
SM: Semimembranosus
BF: Biceps femoris
Q: Quadriceps
RF: Rectus femoris
VL: Vastus lateralis
VM: Vastus medialis
VI: Vastus intermedius
ROI: Region of interest
SD: Standard deviation
ICC: Intraclass Correlation Coefficient
BMI: Body mass index
