# Supplementary material for: Tendon regeneration and muscle hypotrophy after isolated Gracilis tendon harvesting - a pilot study
Source: J Exp Orthop. 2020 Apr 7;7:19. doi: 10.1186/s40634-020-00236-8 (PMC7138873; doi:10.1186/s40634-020-00236-8)
Supplement: Supplementary file 1 — Additional file 1: Supplement 1. Sequences of the MRI of both knees and thighs [file 40634_2020_236_MOESM1_ESM.docx]

**Supplement 1** Sequences of the MRI of both knees and thighs

| **Knee** | **Thigh** |
| --- | --- |
| (acquired unilaterally using a knee coil) | (acquired bilaterally using a surface coil) |
| Proton density fat saturated two-dimensional turbo spin echo sequence in axial, sagittal, and coronal orientation (slice thickness, 3 mm; gap, 0.3 mm) | T1-weighted non-fat saturated two-dimensional spin echo sequence in axial and coronal orientation (slice thickness, 5 mm; gap, 1 mm) |
| proton density weighted non-fat saturated three-dimensional isotropic sequence in sagittal orientation and multiplanar reconstructions (reconstructed slice thickness, 0.6 mm; no gap) | Short tau inversion recovery sequence in coronal orientation (slice thickness, 5 mm; gap, 1 mm) |
|  | T2-weighted half fourier acquired single shot turbo spin echo sequence in sagittal orientation (slice thickness, 5 mm; gap, 1 mm) |
